# Descriptions of a new species, validation of a name, and elevation of a variety in *Iris* (Iridaceae) from China

**DOI:** 10.3897/phytokeys.271.176663

**Published:** 2026-03-16

**Authors:** Zhongzheng Zhang, Gexiang Zhang

**Affiliations:** 1 College of Landscape Architecture, Nanjing Forestry University, Nanjing 210037, China College of Landscape Architecture, Nanjing Forestry University Nanjing China

**Keywords:** Chloroplast genome, Iris, new species, phylogeny

## Abstract

*Iris cariciformis* Z.Z.Zhang & G.X.Zhang, **sp. nov**. from China is described and illustrated. This species grows on cliff faces or open slopes in the central Qinling Mountains. Morphologically, *I. cariciformis* is similar to *I. dabashanensis* but can be distinguished by its longer perianth tube and ellipsoidal capsules with a long beak. Furthermore, this study facilitates the valid publication of *Iris fujianensis*. Phylogenetic analysis based on chloroplast DNA sequences confirms the placement of the former within *I. series Chinenses*, while the latter is inferred as sister to *I. speculatrix*. Simultaneously, a new combination, *Iris valida* (S.S.Chien) Z.Z.Zhang, H.T.Li & T.Y.Zuo, **comb. et stat. nov**., is proposed.

## Introduction

The genus *Iris* L. (s.l.) is the most species-rich genus within the family Iridaceae Juss., comprising approximately 300 species (Zhao et al. 2000; Manning and Goldblatt 2008). Plants of this genus not only possess high ornamental value, leading to the development of numerous horticultural cultivars, but some species also hold significant medicinal importance (Khatib et al. 2022). As a genus widely distributed across the temperate Northern Hemisphere, *Iris* exhibits considerable morphological variation. Taxonomists have long held differing views regarding the circumscription of the genus and the establishment of its infrageneric categories (Rodionenko 1961; Crespo et al. 2015).

*Iris series Chinenses* (Diels) Lawrence is a small group of taxa that occur in East Asia, characterized by distinct identifying features: creeping and slender rhizomes, prominent leaf veins, falls with a low undulate or nearly entire ridge, and seeds with horn-like appendages (Lawrence 1953; Mathew 1989). Under the circumscription by Mathew (1989), this series was defined to include *I. speculatrix* Hance, which is widely distributed in southern China. In contrast, based on its prominently crested sepal appendages, Dykes (1913) and Lawrence (1953) placed this species in *I.ser. Evansia* (Alef.) Baker and *I. subsect. Evansia* (Alef.) Benth. & Hook.f., respectively. However, *I. speculatrix* is characterized by tall plants, large flowers, thick rhizomes with stout, sparsely branched roots (Zhao et al. 2000). Furthermore, multiple phylogenetic studies based on chloroplast genes have indicated that it does not belong to this series (Tillie et al. 2000; Guo and Wilson 2013; Siu et al. 2022). Additionally, Su (2024) proposed the name “*I. fujianensis* X.X.Su” for a new species discovered in Fujian Province, China, noting its morphological similarity to *I. speculatrix*. Although the differential characters outlined in the abstract against *I. speculatrix* could potentially be treated as a valid English diagnosis, the name was nevertheless deemed invalid because a holotype was not designated and the cited material comprised 2 separate gatherings, in non-compliance with Art. 8.2 and 40.1 of the “International Code of Nomenclature for algae, fungi, and plants” (Turland et al. 2018). Crespo et al. (2025) identified *Iris speculatrix* as the sister group to the *Xiphion* clade and proposed the new genus *Portiodora* M.B.Crespo, Mart.-Azorín & Mavrodiev to accommodate this species. However, due to the absence of key morphological features and molecular data, *I. fujianensis* was not included within *Portiodora*.

Within *I. ser. Chinenses*, a total of 8 taxa were recognized by Crespo et al. (2015) in the same taxonomic treatment where they proposed the separate genus *Zhaoanthus* M.B.Crespo, Mart.-Azorín & Mavrodiev. Excluding *I. speculatrix*, the remaining species included *I. henryi* Baker, *I. odaesanensis* Y.N.Lee, *I. minutoaurea* Makino, *I. koreana* Nakai, *I. rossii* Baker, and *I. proantha* Diels along with its variety *I. proantha var. valida* (S.S.Chien) Y.T.Zhao. Wilson (2020) later described 2 additional species from central China, *I. dabashanensis* C.A.Wilson and *I. probstii* C.A.Wilson, thereby further expanding the knowledge of this series. Notably, during our compilation of resources and literature review on *I. ser. Chinenses*, we observed that *I. proantha var. valida* exhibits taller plants, larger flowers, and shows distinct distribution patterns (Zhao et al. 2000), suggesting that it may represent an independent species.

During a plant resource survey conducted by our research team in the Qinling Mountains, a morphologically distinct taxon of the genus *Iris*, belonging to *I. ser. Chinenses*, was discovered. Detailed morphological comparisons revealed significant differences in its floral structure and fruit morphology from all known species within the series suggesting that it likely represents a new species. Chloroplast genomes provide comprehensive resolution of complex phylogenetic relationships among closely related species, offering far more informative sites for phylogenetic analysis than traditional standard DNA fragments. This significantly enhances both the resolution and statistical support of phylogenetic trees, thereby effectively revealing the evolutionary history among taxa. This study integrates morphological evidence with chloroplast genome data to clarify the relationships of this newly discovered species and “*I. fujianensis*” with their respective closely related taxa. Additionally, this study validates the name “*I. fujianensis*” and clarifies the taxonomic relationship between *I. proantha* and its variety, *I. proantha var. valida*. The findings will contribute to a more robust classification system for the genus *Iris* and provide a scientific basis for the conservation and sustainable utilization of *Iris* diversity in China.

## Materials and methods

### Morphological study

The morphological study in this research was primarily based on living field materials and images of specimens from herbarium collections. Relevant specimen images were mainly sourced from the Chinese Virtual Herbarium (CVH; https://www.cvh.ac.cn/), the Global Biodiversity Information Facility (GBIF; https://www.gbif.org/), and the Integrated Digitized Biocollections (iDigBio; https://www.idigbio.org/). During the study, morphological observations and corresponding measurements were conducted on roots, rhizomes, leaves, bracts, flowers, and fruits.

### Phylogenetic analysis

All 6 samples used in this experiment were collected from the wild and cultivated in the nursery. Sample information is listed in Suppl. material 1. Fresh leaves were dried with silica gel, after which total DNA was extracted using the CTAB method (Doyle and Doyle 1987). After quantifying and assessing the quality of the DNA, library preparation and sequencing were performed following the standard Illumina protocol. The above procedures were conducted by Wuhan Benna Technology Co., Ltd.

The resulting paired-end reads were approximately 150 bp in length. Low-quality reads and adapter sequences were removed using FastQC v0.11.5 (http://www.bioinformatics.babraham.ac.uk/projects/fastqc/). After quality control, the plastomes were assembled automatically using GetOrganelle v1.7.7.1 (Jin et al. 2020) and annotated with CPGAVAS2 (Shi et al. 2019), followed by manual adjustments. For annotation, plastid sequences from *I. odaesanensis* (NC_056178.1), *I. speculatrix* (PP734292.1), and *I. japonica* Thunb. (NC_060499.1) were used as references.

Phylogenetic trees were constructed based on 44 chloroplast genome sequences from *Iris* and *Moraea* Mill., including 6 newly sequenced during this study. Complete plastome DNA sequences (excluding one inverted repeat) were aligned using MAFFT v7.525 (Katoh and Standley 2013). Following Kamra et al. (2023), *Moraea spathulata* (L.f.) Klatt and *M. polystachya* (Thunb.) Ker Gawl. were designated as outgroups for phylogenetic analysis using maximum likelihood, maximum parsimony, and Bayesian inference methods.

The best-fit model (K3Pu+F+R4) was selected based on the Bayesian Information Criterion (BIC) using ModelFinder v2.2.0 (Kalyaanamoorthy et al. 2017). Subsequently, the maximum likelihood (ML) phylogenetic tree was reconstructed using IQ-Tree v3.0.1 (Nguyen et al. 2015) under this model, with 1000 rapid bootstrap replicates to estimate bootstrap support values for nodes. Maximum parsimony analysis was performed using MEGA v11 (Tamura et al. 2021). Heuristic searches were conducted with 100 random addition sequence replicates followed by Tree-Bisection-Reconnection (TBR) branch swapping at search level 2. Branch support was evaluated with 1000 standard bootstrap pseudoreplicates. Bayesian inference was performed using MrBayes v3.2.7 (Ronquist et al. 2012). For Bayesian inference, we employed the standard GTR+I+G model, which is the most general model available in MrBayes and suitably accommodates the evolutionary characteristics identified by model selection. Markov chain Monte Carlo (MCMC) simulations were conducted with 2 independent runs for 5 million generations, sampling trees every 1,000 generations. The first 25% of trees were discarded as burn-in

## Results

### Morphological comparisons

The subterranean morphology of *I. cariciformis* is relatively consistent with members of *I. ser. Chinenses*, characterized by slender, elongated rhizomes and fine fibrous roots, with root nodules occasionally observed. In terms of aboveground morphology, this species closely resembles *I. dabashanensis*, notably sharing a leaf width of only 2–3 mm, which distinguishes them significantly from other members of *I. ser. Chinenses*. The primary morphological differences between *I. cariciformis* and *I. dabashanensis* are found in the floral structures: *I. cariciformis* possesses a short pedicel and a long perianth tube, whereas *I. dabashanensis* exhibits a long pedicel and a short perianth tube. Furthermore, the capsule of *I. cariciformis* is ellipsoid with a long beak, whereas that of *I. dabashanensis* is globose and lacks a beak. The seed morphology of *I. cariciformis* is consistent with other members of *I. ser. Chinenses*, characterized by an ellipsoid shape with corniculate appendages.

*Iris rossii*, *I. proantha*, and *I. valida* (= *I. proantha var. valida*) are morphologically similar, sharing a slender perianth tube and globose fruits. However, they differ in leaf dimensions (length and width), stem height, and perianth tube length. Beyond these distinctions, the 2 species also differ in seed morphology: seeds of *I. proantha* are nearly globose with inconspicuous appendages, whereas those of *I. valida* are irregularly globose with prominent white, horn-shaped appendages. Detailed comparisons are presented in Table 1.

**Table 1.** Morphological traits of *Iris rossii*, *I. proantha* and *I. valida*.

Character	*I. rossii*	*I. proantha*	*I. valida*
Leaf (at anthesis)	4–10 (15) × 0.2–0.5 cm	5–20 × 0.1–0.25 cm	Ca. 27 × ca. 0.7 cm
Leaf (in fruit)	Not specified	40 cm × 7 mm	55 cm × ca. 8 mm
Scape Height	Very short	5–7 cm	20–28 cm
Bract Length	4–7 cm	3.5–5.5 cm	4.3–5.5 cm
Flower Color	Bluish–purple	Pale bluish–purple	Pale bluish–purple
Flower Diameter	3.5–4 cm	3.5–4 cm	ca. 5 cm
Perianth Tube Length	5–7 cm	2.5–3 (5) cm	3–6 cm
Sepals	Ca. 3 × 0.8**–**1.2 cm	Ca. 2.5 × 1–1.2 cm	Ca. 2.6 cm × ca. 10 mm
Petals	Ca. 2.5 × 0.8 cm	2.2–2.5 × ca. 0.7 cm	2–2.2 × ca. 0.8 cm
Stamen Length	Ca. 1.5 cm	Ca. 1 cm	Ca. 7 mm
Style Branch Length	Ca. 2 cm	Ca. 1.8 cm	Ca. 1.6 cm
Ovary Length	Ca. 1 cm	Ca. 4–5 mm	Not described
Fruit	Capsule, globose,	Capsule, globose, with a short beak at apex	Capsule, globose
Seed	Irregularly globose, with white, horn-shaped appendages	Irregularly globose, with horn-shaped appendages	Nearly globose, appendages inconspicuous
Flowering Period	April–May	March–April	April
Fruiting Period	June–August	May–July	May–July
Distribution	Southern Liaoning, Korea, and southern Japan	Henan, central Anhui, southern Jiangsu, northern Jiangxi, and central and central–western Hubei	Northern Zhejiang and southern Anhui

The morphological characteristics shared by *I. fujianensis* and *I. speculatrix* are as follows: a relatively thick and obliquely ascending rhizome; thick, stout, and sparsely branched roots; a thick and short perianth tube; low and entire crest-like sepal appendages; long-beaked fruits; and irregular polyhedral seeds with appendages. In addition, both *I. fujianensis* and *I. speculatrix* have reduced leaves at the base of the flowering stem (this feature is not observed in some specimens of *I. speculatrix*). The leaves of *I. fujianensis* are longer and wider than those of *I. speculatrix*. Significant floral differences also exist between the 2 species: *I. fujianensis* produces a single flower per scape, with a flower diameter of 10–12 cm, whereas *I. speculatrix* bears 2 flowers per scape, each with a diameter of only 5.6–6 cm. Furthermore, the 2 species differ in floral patterning: the sepals of *I. fujianensis* have a white background adorned with irregular blue lines and a blue apex, while those of *I. speculatrix* are bluish-purple with a distinct white horseshoe-shaped marking in the center and bluish-purple stripes on the claw section.

### Phylogenetic relationships

The chloroplast genomes of all 6 newly sequenced *Iris* taxa (among which 2 were newly confirmed) exhibited the typical quadripartite structure – comprising a pair of inverted repeat (IR) regions, a large single-copy (LSC) region, and a small single-copy (SSC) region.

**Figure 1.**
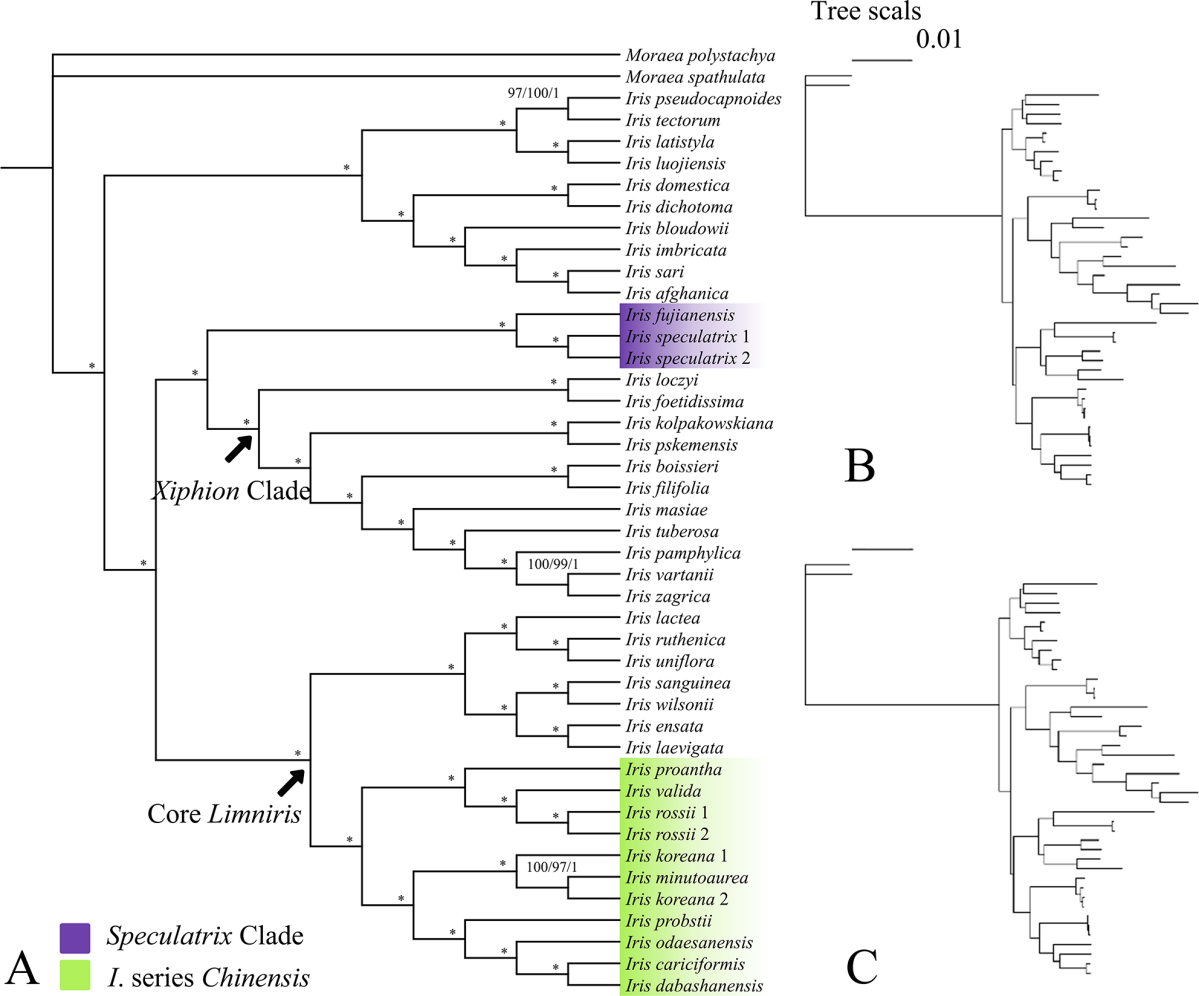
Phylogenetic analysis of *Iris* species based on chloroplast genomes. **A**. The tree shape congruent between maximum likelihood (ML), maximum parsimony (MP), and Bayesian inference (BI) analyses. The numbers above the branches represent bootstrap support values from ML (left) and MP (middle) analyses, and Bayesian posterior probabilities (right), respectively. An asterisk indicates nodes with maximum support (ML/MP BS = 100; BI PP = 1); **B**. The ML tree with branch lengths; **C**. The BI tree with branch lengths.

The maximum likelihood, maximum parsimony, and Bayesian trees constructed using the chloroplast genome dataset showed congruent topologies (Fig. 1). The results support the monophyly of the “Core *Limniris*” clade as defined by Wilson (2011). Within this clade, *I. ser. Chinenses* is the earliest diverging lineage and is sister to all remaining members. The internal phylogenetic structure of *I. ser. Chinenses* is as follows: the group composed of *I. proantha*, *I. valida*, and *I. rossii* is sister to the remaining species within the series. Within this group, *I. valida* and *I. rossii* are sister to each other, while *I. proantha* is not monophyletic with *I. valida* but instead is sister to the clade containing *I. valida* + *I. rossii*. Additionally, *I. koreana* was not recovered as monophyletic, with *I. minutoaurea* nested within it; together they form a strongly supported clade. The sister relationship between *I. cariciformis* and *I. dabashanensis* is strongly supported, and the clade they form is sister to *I. odaesanensis*. Finally, *I. probstii* is sister to the entire clade comprising *I. odaesanensis* + (*I. cariciformis* + *I. dabashanensis*).

The sister relationship between *I. speculatrix* and *I. fujianensis* is strongly supported. The clade formed by these 2 species, the *Speculatrix* Clade, is independent of *I. ser. Chinenses* and is sister to the *Xiphion* Clade.

### Taxonomy

#### Iris cariciformis

Taxon classificationPlantaeAsparagalesLycaenidae

Z.Z.Zhang & G.X.Zhang sp. nov.

2D5198E1-B423-55B9-A4FE-B3558B708BC5

urn:lsid:ipni.org:names:77377783-1

Fig. 2

##### Diagnosis.

Morphologically similar to *I. dabashanensis*, but differs by the longer perianth tube (1.6–2.5 cm vs 0.2–0.5 cm) and ovoid capsules with a prominent beak (vs globose and beakless).

**Figure 2.**
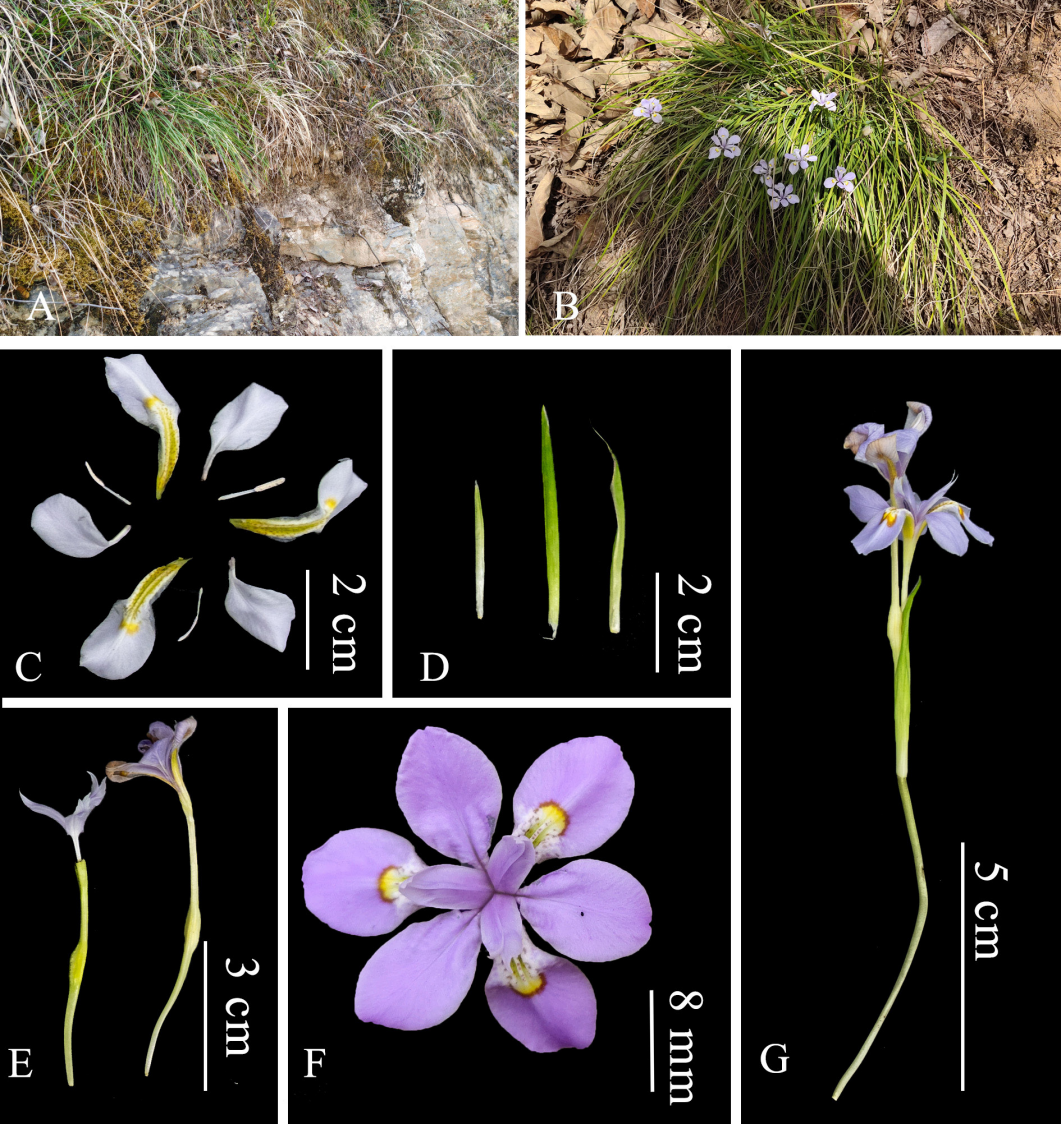
*Iris cariciformis* Z.Z.Zhang & G.X.Zhang, sp. nov. **A**. Habitat; **B**. Plant; **C**. Sepals, petals and stamens; **D**. Bracts; **E**. Pedicel, ovary and pistil; **F**. Flower; **G**. Inflorescence (Photos A, B, C. author; C–G Xiaoqiang Shen).

##### Type.

China • Shaanxi Province, Zhashui County, Liushugou, on the dry hillside; 326 m; 33°40'09.8"N 109°10'50.3"E; 30 March 2025 (fl); *Z.Z.Zhang ZZZ-LSG-001* (**holotype**: CSH!; **isotypes**: CSH!, NAS!).

##### Description.

Perennial tufted herb with persistent fibrous leaf remnants sheathing the base. Rhizomes slender, usually stoloniferous. Roots slender, much branched, with small root nodules. Leaves pale green, narrowly linear, 20–40 cm × 2–3 mm, with 1 or 2 veins. Flowering stems 10–15 cm tall, bearing 1–2 leaves; bracts 2 (rarely 3), green, narrowly lanceolate, 2.7–4.2 cm × ca. 4 mm. Flowers pale purple, 2.5–3 cm in diameter; pedicels slender, 1.7–2.5 cm long; perianth tube slender, 1.6–2.5 cm long; sepals pale purple, horizontal and recurved distally, broadly ovate, 1.8–2.3 cm × 6–9 mm, base narrowed and yellow, midvein creamy white with purple spots, entire, with 2 irregularly raised lateral ridges; petals slightly angled upward, suborbicular, 1.5–1.7 cm × ca. 7 mm, abruptly contracted into a claw at the basal quarter. Stamens creamy white, ca. 8 mm long; anthers linear, equal to filaments; ovary ca. 6 mm long; style branches ascending, ca. 1.3 cm × 4 mm, apex bifid with lobes ca. 4 mm long. Capsule ovoid with a prominent beak, 1.7–2 cm × ca. 1 cm. Seeds brown, 2–3 mm in diameter, with white appendages. Flowering April, fruiting May–June.

##### Distribution and ecology.

**Figure 3.**
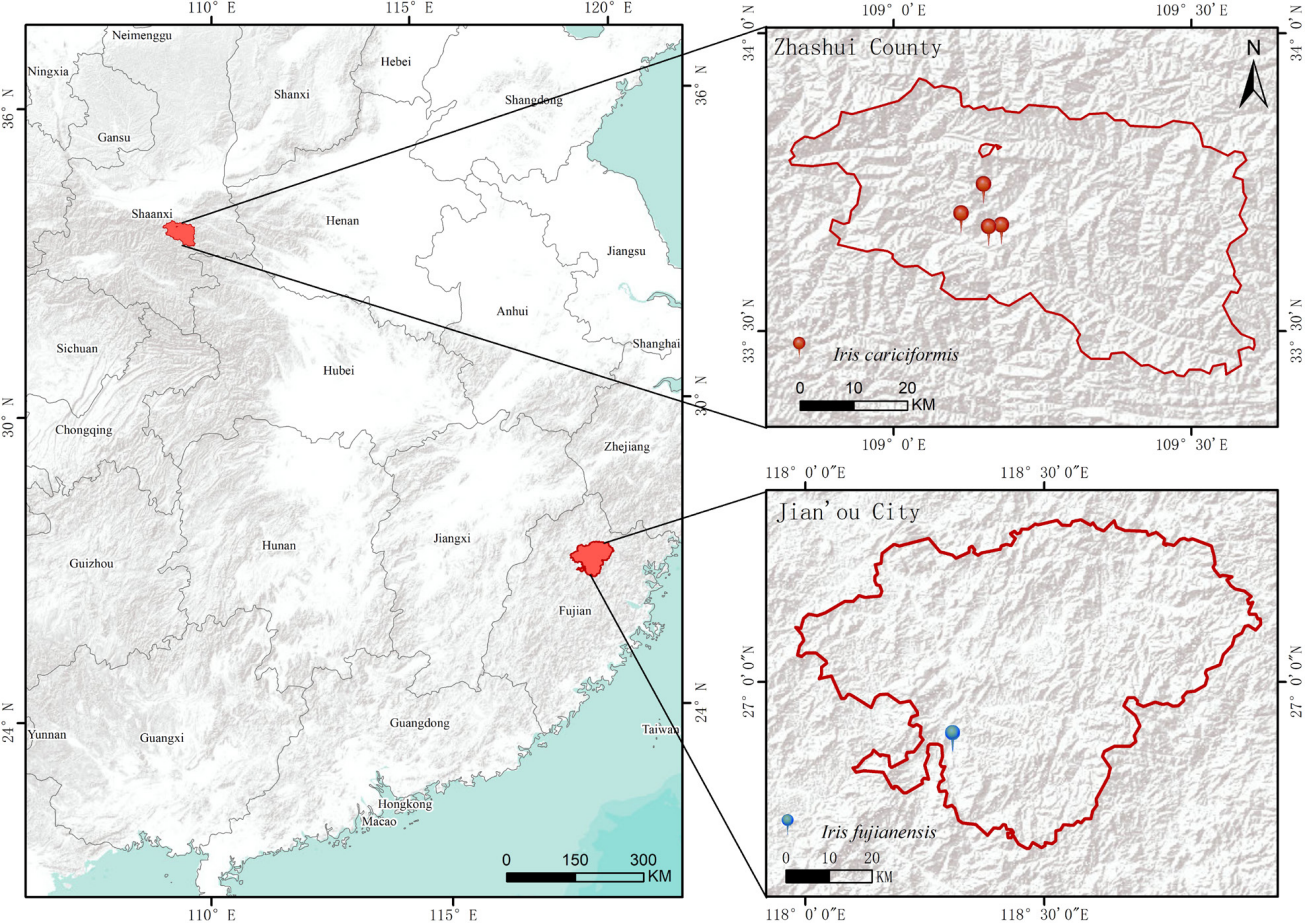
The known distribution of *Iris cariciformis* and *I. fujianensis* in China.

*Iris cariciformis* is distributed in the central Qinling Mountains (Zhashui County, Shaanxi Province, China), where it grows on mountain slopes and cliffs (Fig. 3).

##### Etymology.

The new species grows in clusters and has slender leaves, resembling plants of the *Carex* L. that inhabit the same environment, hence the specific epithet “cariciformis” was chosen.

##### Preliminary conservation status.

According to the Red List Categories and Criteria of IUCN (2024), the conservation status of *I. cariciformis* is preliminarily assessed as Least Concern (LC). This species meets the criteria for the LC category due to its relatively wide distribution, considerable population size, and intact population structure (including both seedlings and mature individuals).

#### Iris fujianensis

Taxon classificationPlantaeAsparagalesLycaenidae

X.X.Su

90F3BC91-8940-567B-B881-FD67BF6392C6

urn:lsid:ipni.org:names:77377792-1

##### Invalid synonym.

*Iris fujianensis* X.X.Su in J. Fujian For. Sci. Technol. 51 (3): 101–103. 2024 — invalid name (Art. 8.2, 40.1 of ICN); holotype not indicated; 2 gatherings included.

##### Diagnosis.

*Iris fujianensis* is morphologically similar to *I. speculatrix*, but differs by having longer and wider leaves (20–80 × 1.4–2.6 cm vs. 15–30 × 0.6–1.2 cm), shorter pedicels (1–2 cm vs. 3–5.5 cm), and solitary flowers with a larger diameter (10–12 cm vs. 5.6–6 cm).

##### Type.

China • Fujian Province, Jian’ou City, Yushan, located on both sides of the damp mountain stream. 326 m, 26°52'08.4"N 118°16'42.1"E, 4 May 2022 (fr), *X.X.Su SXX0077* (*holotype*: CSH!)(lectotype, designated here). Same locality as for holotype, 11, April, 2023 (fl), *X.X.Su XY02916* (*paratype*: CSH!).

##### Description.

Perennial herb, base with fibrous remnants of old leaf sheaths. Rhizome stout, yellowish-brown, 1–1.5 cm in diam. Leaves deep green, broadly ensiform, 20–80 × 1.4–2.6 cm, with several inconspicuous longitudinal veins, apex acuminate, base sheathing. Flowering stem 10–25 cm tall, erect, simple, bearing 3–5 reduced leaves at base; bracts 2, green, herbaceous, margin membranous, lanceolate, 7–8 × 1.5–2.5 cm, apex long-acuminate. Inflorescence with a single flower. Flowers pale blue, 10–12 cm in diam; pedicel 1–2 cm long; perianth tube funnelform, ca. 1 cm long; sepals spreading, spatulate, ca. 8 × 2–2.5 cm, white with blue stripes, apex obtuse, claw cuneate, bearing a prominent yellow crested appendage; petals long-spatulate, 7–8 × ca. 2 cm, spreading, upper part pale blue, lower part white with blue stripes; stamens ca. 3.5 cm long; anthers white, subequal to filament; style branches flat, pale blue, ca. 6.5 cm long, apical lobes deltoid, margin incised; ovary ca. 1.8–2 cm long. Capsule trigonous-ellipsoid, 4–6 × 2–2.5 cm. Capsule trigonous-ellipsoid, 4–6 × 2–2.5 cm, with a slender, pointed beak at the apex, pedicel not curved after flowering. Seeds irregularly polyhedral, ca. 3 mm in diam., with transparent appendages. Flowering April, fruiting May–August.

##### Distribution and habitat.

This species is currently known only from the type locality on Yushan in Jian’ou City, Fujian Province, China. It grows on shaded, damp slopes along mountain streams (Fig. 3).

##### Other specimens examined.

Same locality as for holotype, 11 April 2023 (fl), XY02917 (NAS!)

##### Notes.

*Iris fujianensis* was not validly published as a holotype was not designated and the material comprised 2 separate gatherings. To validate the name, we designate a specimen from the material cited by Su (2024) as a lectotype (serving here as the holotype). Concurrently, the description and diagnosis provided by Su (2024) were compiled as above.

#### Iris valida

Taxon classificationPlantaeAsparagalesLycaenidae

(S.S.Chien) Z.Z.Zhang, H.T.Li & T.Y.Zuo comb. et stat. nov.

F85E09A0-3EA7-543F-BC91-FA0D28B3FFFD

urn:lsid:ipni.org:names:77377790-1

##### Basionym.

*Iris pseudorossii var. valida* S.S.Chien, Contr. Biol. Lab. Sci. Soc. China, Bot. Ser. 6: 74. 1931.

##### Type.

China • Chekiang [Zhejiang] Province, W. Tien-mu-shan, under woods. 18, April 1931, *W.C.Cheng 2361* (*holotype*: location unknown) · E. tien-mu-shan, on open slopes and roadsides, 14 April 1931 *W.C.Cheng 2246 (paratypes*: PE 00034036, IBSC 0628903)

##### Homotypic synonyms.

*Iris proantha var. valida* (S.S.Chien) Y.T.Zhao, Acta Phytotax. Sin. 20 (1): 100. 1982. *Zhaoanthus proanthus var. validus* (S.S.Chien) M.B.Crespo, Mart.-Azorín & Mavrodiev, Phytotaxa 232 (1): 60. 2015.

## Discussion

In the genus *Iris*, the sepal crest is diverse in form, and species possessing this feature are distributed across multiple independently evolved lineages (Guo 2015; Guo and Wilson 2013). The 2 crested taxa discovered in this study were found to be closely related to members of *I. ser. Chinenses* and *I. speculatrix*, respectively.

*Iris cariciformis* can be distinguished from members of *I. ser. Chinenses* by characteristics such as leaf blade width, pedicel and perianth tube length, and fruit morphology. The similarity in its vegetative organs to those of *I. dabashanensis* suggests a close relationship between them, which is confirmed by the phylogenetic analysis in this study. Through the examination of specimens and literature records of *I. dabashanensis*, it has been found that its distribution range is wider than initially inferred by Wilson (2020) in the original description, extending westward to the Xiaolongshan area in the western Qinling Mountains (Huang et al. 2022). The distribution of *I. cariciformis* is currently known only within Zhashui County, Shaanxi Province, and its range does not overlap with that of *I. dabashanensis*. It is inferred that the differentiation between *I. cariciformis* and *I. dabashanensis* resulted from geographic isolation caused by the orogenic activities of the Qinling and Daba Mountains.

Furthermore, as a significant dividing line between the warm temperate and subtropical zones, the Qinling region exhibits high species richness. However, records of *Iris* species in this area are relatively scarce, necessitating more extensive and detailed surveys. Unfortunately, we were unable to obtain samples of *I. henryi* to clarify its phylogenetic position within *I. ser. Chinenses*.

*Iris proantha* and *I. valida* are morphologically similar. However, *I. valida* is distinguished by its taller scape, larger flowers, and longer and wider leaves. Chien and Cheng (1931) noted that it was unclear whether these differences were due to environmental factors and mentioned that no intermediate forms had been observed. Nevertheless, key taxonomic evidence comes from seed morphology – a relatively stable, genetically adapted trait less influenced by environmental variation. The conspicuous white, horn-shaped appendages on the seeds of *I. valida* and *I. rossii* may serve to attract ants or other insects for dispersal. In terms of distribution, *I. valida* occurs in northern Zhejiang and southern Anhui, inhabiting open slopes or forests at higher elevations, and its distribution range does not overlap with that of *I. proantha*. Furthermore, our phylogenetic results show that *I. valida* and *I. proantha* do not form a monophyletic group, which does not support the taxonomic treatment of *I. valida* as a morphological variant of *I. proantha* (Zhao 1982).

All species of *I. ser. Chinenses* are distributed in East Asia, yet their geographic distributions exhibit significant variation. *I. proantha* and *I. valida* occur in the mountainous and hilly areas of eastern China, while *I. henryi*, *I. probstii*, *I. dabashanensis*, and *I. cariciformis* are found across a broad region from the Qinling Mountains to the Wuling Mountains in central China. *I. minutoaurea*, *I. odaesanensis*, and *I. rossii* are primarily distributed on the Korean Peninsula and adjacent areas of northeastern China, with only *I. rossii* extending to central and southern Japan (Naito and Nakagoshi 1995). *I. koreana* is restricted to the central and southern parts of the Korean Peninsula (Pi et al. 2016). Phylogenetically, the species distributed on the Korean Peninsula and northeastern China, as well as those in central and eastern China, do not form separate geographically defined monophyletic groups, suggesting that this series has undergone multiple dispersal events. Relevant studies have indicated that the flora of central and eastern China shares greater similarity with that of the Japanese archipelago and the Korean Peninsula, which is confirmed in the case of *I. ser. Chinenses*. Additionally, it is noteworthy that Park et al. (2022) inferred an allotetraploid hybrid origin for *I. koreana* from *I. odaesanensis* and *I. minutoaurea*, with *I. odaesanensis* likely serving as the maternal donor. This hypothesis explains the observed similarity in plastid markers between *I. odaesanensis* and the 2 *I. koreana* samples.

The phylogenetic analysis in this study further confirms previous conclusions that *I. speculatrix* does not belong to *I. ser. Chinenses* (Tillie et al. 2000; Guo and Wilson 2013). Moreover, by including *I. fujianensis* for the first time, it reveals that these 2 species form a strongly supported clade, which is itself resolved as the sister group to the *Xiphion* Clade. The placement of *I. fujianensis* outside the *Speculatrix* Clade by Crespo et al. (2025), based on morphological evidence, may be attributed to an oversight of similarities in their vegetative organs and the absence of key morphological characters from the original description by Su (2024), such as an entire stylar lip and seeds bearing appendages. It is worth noting that the description and illustrations by Su (2024) depict a “stalk-like” structure on the seeds of *I. fujianensis*; this structure is actually a seed appendage that shrivels upon drying but remains clearly visible.

*Iris fujianensis* is distinctly different from *I. speculatrix* in its unique morphology – specifically, its longer and wider leaves, single and larger flower, and shorter pedicel. Reduced leaves are present at the base of the flowering stem in *I. fujianensis*. Similar structures have also been observed in some specimens of *I. speculatrix*, though they are absent in others. According to Wilson (2020), the presence or absence of reduced leaves at the base of the flowering stem is an important characteristic distinguishing *I. speculatrix* from the 2 taxa long treated as its synonyms *– I. grijsii* Maxim. and *I. cavaleriei* H. Lév. More detailed population studies are needed to clarify whether this feature is stable among these species.

## Supplementary Material

XML Treatment for Iris cariciformis

XML Treatment for Iris fujianensis

XML Treatment for Iris valida

## Additional information

### Use of AI

No use of AI was reported.

### Author ORCIDs

Zhongzheng Zhang https://orcid.org/0009-0000-0873-6440

### Data availability

All of the data that support the findings of this study are available in the main text or Supplementary Information.

## Supplementary materials

Supplementary material 1 Accession of 38 sequenced or downloaded chloroplast genome in this study Data type xlsx Explanation note Owing to current processing delays at NCBI, we have been unable to obtain an Accession Number prior to submission. We are submitting the data to an alternative repository and will provide the necessary accession details prior to the final publication of the manuscript to ensure full compliance with data availability policies. https://binary.pensoft.net/file/1562761This dataset is made available under the Open Database License (http://opendatacommons.org/licenses/odbl/1.0/). The Open Database License (ODbL) is a license agreement intended to allow users to freely share, modify, and use this Dataset while maintaining this same freedom for others, provided that the original source and author(s) are credited. Zhongzheng Zhang, Gexiang Zhang
